# Inter-Session Reliability of an Isometric Muscle Strength Protocol in Older Adults

**DOI:** 10.3390/jfmk11010031

**Published:** 2026-01-09

**Authors:** Oscar Andrades-Ramírez, Domingo Ceballos-Sepulveda, Benjamín Fuentealba-Martínez, Benjamín Venegas-Cofré, Víctor Valenzuela-Zieballe, Humberto Castillo-Quezada, Bryan Alfaro-Castillo, Luis Romero-Vera, Claudio Carvajal-Parodi, Claudio Hernández-Mosqueira

**Affiliations:** 1 Facultad de Educación y Ciencias Sociales, Universidad Andres Bello, Concepción 4070387, Chileb.fuentealbamartinez@uandresbello.edu (B.F.-M.); b.venegascofr@uandresbello.edu (B.V.-C.); v.valenzuelazieballe@uandresbello.edu (V.V.-Z.); humberto.castillo@unab.cl (H.C.-Q.); 2 Departament of Sports Sciences and Physical Conditioning, Universidad Católica de la Santísima Concepción, Concepción 4090541, Chile; 3 Facultad de Humanidades y Educación, Universidad de Atacama, Copiapó 1531772, Chile; bryan.alfaro@uda.cl; 4 Facultad de Salud y Ciencias Sociales, Universidad de las Américas, Concepción 4030000, Chile; luis.romero.vera@edu.udla.cl; 5 Escuela de Kinesiología, Facultad de Ciencias de la Rehabilitación y Calidad de Vida, Universidad San Sebastián, Concepción 4030000, Chile; claudio.carvajal@uss.cl; 6 Departamento de Ciencias de la Educación, Facultad de Educación y Humanidades, Universidad del Bio-Bio, Chillán 3800708, Chile

**Keywords:** muscle strength assessment, isometric muscle strength, reliability, older adults

## Abstract

**Background:** The objective of this study was to analyze the absolute and relative reliability intersession for a maximal isometric muscle strength protocol in the bilateral seated bench press (BSBP), bilateral seated row (BSR), unilateral seated knee right extension (USKER) and unilateral seated knee left extension (USKEL) in a population of older adults. **Methods**: Eighteen older adults (age = 69.38 ± 5.06 years; weight = 75.79 ± 14.18 kg; height = 1.61 ± 0.08 m; BMI = 28.98 ± 5.04 kg/m^2^. The maximal isometric muscle strength assessment was performed in a seated position. Participants were asked to exert maximum effort during the exercise. The BSBP and BSR assessments were performed bilaterally with shoulders and elbows at 90°, while the USKER and USKEL assessments were performed unilaterally. Three sets of 5 secondswere performed with a 3 minutes rest between measurements until maximum isometric strength was reached in all four measurements. **Results**: In the inter-session reliability measurements, acceptable absolute reliability was presented for BSR and USKER, and extremely high reliability for the BSBP and USKEL measures. In addition, extremely high relative reliability was reported for all assessments of maximum isometric muscle strength, with no significant differences were observed (*p* > 0.05) and an ES classified as null (ES < 0.12). **Conclusions**: The main results of this study show that maximal isometric muscle strength in bilateral seated bench press, bilateral seated row, and unilateral seated right and left knee extension, assessed using the Chronojump Force Sensor Kit, is reliable and reproducible for the elderly population.

## 1. Introduction

Human aging is a process characterized by a gradual decline in physiological functions [1]. This is associated with an increased prevalence of physical disability, risk of falls, and the possibility of musculoskeletal injuries [2]. Several variables contribute to muscle loss in older adults, such as the physiological process of aging itself [3], inadequate nutrition, and a sedentary lifestyle [4]. Among the main physiological changes reported in older adults is the decrease in muscle mass, which leads to a progressive decline in the ability to generate muscle force [5]. Muscle strength, in addition to all the locomotor benefits it provides to older adults, is a determining factor in independence in this population, due to its influence on the physical functioning of individuals, which can lead to an inability to perform activities of daily living or a loss of functional independence [6]. In this sense, having accurate and reproducible assessments when evaluating muscle strength and monitoring strength is essential for healthcare personnel when performing any preventive or physical rehabilitation intervention [7,8].

There are various methods to evaluate and measure the different manifestations of muscular strength, such as dynamic strength, isotonic strength, variable resistance, and strength assessment based on mass displacement velocity [9]. One of the muscle strength assessments of great interest in the scientific world is isometric assessment, which is a simple method at the time of assessment and the equipment is inexpensive [10]. Previous studies have shown that isometric muscle strength assessment presents a low risk of injury due to its ability to isolate specific muscle groups [11]. Among the advantages over other strength measurement tests, such as dynamic tests, it has been found that isometric contractions provide more standardized records by eliminating the influence of speed and execution technique [12]. Its methodological simplicity makes this type of assessment a very attractive alternative for assessing muscle strength in clinical populations and older adults [13].

Despite all its reported advantages [14,15], the application of isometric tests with load cells presents challenges related to the reliability of the instruments used [16,17], as well as the absence of standardized methods for implementing protocols in different types of populations [18]. Isometric strength assessments performed on older adults, where observed muscle strength may be small in a statistical context but generate clinically significant changes, it is essential to evaluate the reliability of both the protocols and the muscle strength assessment devices [5,19]. Current scientific evidence reports various studies using hand dynamometers, force platforms, and load cells to assess isometric strength [20,21].

However, there is still a gap in the application of these maximum muscle strength assessments, with young people, athletes, and patients with specific pathologies being the most studied, leaving a large gap in our knowledge when we want to implement these methodologies in older adults [8,22,23]. The absence of standardized protocols limits the applicability of muscle strength assessment results. Because of this, it is essential to analyze the absolute and relative reliability of a protocol that assesses maximum isometric muscle strength between sessions in older adults, which would favor the inclusion of this type of test in clinical practice and in future exercise prescriptions in older adult populations.

Therefore, the objective of this study was to analyze the absolute and relative intersession reliability of a maximal isometric muscle strength assessment for a maximal isometric muscle strength protocol in the bilateral seated bench press (BSBP), bilateral seated row (BSR), unilateral seated knee right extension (USKER) and unilateral seated knee left extension (USKEL) in a population of older adults.

## 2. Materials and Methods

### 2.1. Study Design

A repeated measures design was used to analyze the absolute and relative reliability of an isometric maximal muscle strength test for BSBP, BSR, USKER, and USKEL using the Chronojump force sensor kit (Chronojump, BoscoSystem, Barcelona, Spain). Participants attended two familiarization sessions (at least 72 h apart). The first session (a) assessed anthropometry and (b) familiarized the participants with the procedures and measurement instrument. Subsequently, participants began experimental strength assessment trials on different days. Assessments were performed in the sports and exercise sciences laboratory of Universidad Andres Bello, Concepción, Chile. All assessments were performed at the same time of day (±1 h) for each participant.

### 2.2. Participants

Eighteen older adults (male = 6; female = 12; age = 69.38 ± 5.06 years; weight = 75.79 ± 14.18 kg; height = 1.61 ± 0.08 m; BMI = 28.98 ± 5.04 kg/m^2^), with no experience in maximum isometric strength assessments, physically active, self-sufficient older adults, voluntarily participated in the study. Before giving their written consent, each participant was informed about the purpose, nature, and risks of the study assessment technique. Participants who took part in this study (a) did not have serious musculoskeletal diseases that affected muscle strength and (b) followed verbal instructions from the evaluators. Participants who did not complete the two assessment sessions were excluded. The Ethics and Research Committee of Andrés Bello University, code 06/2024, approved the study protocol on 12 December 2024. All procedures performed in this study were conducted in accordance with the Declaration of Helsinki [24].

### 2.3. Materials

The assessment of maximum isometric strength was performed with Chronojump force sensor kit (Chronojump, BoscoSystem, Barcelona, Spain). This device has a maximum load capacity of 500 kg, an analog-to-digital converter with 24-bit resolution, and a frequency of 160 Hz, RCA trigger input. The sensor connects via a computer with a USB port and works in conjunction with the Chronojump Boscosystem software version 2.46, which provides a real-time visualization of the applied force, as well as storing and analyzing the data obtained. Its main parameters include peak force, rate of force development (RFD), time to peak force, and the complete force-time curve.

### 2.4. Assessment of Peak Muscle Strength

A familiarization phase of the evaluation was carried out, which consisted of two sessions. The first session consisted of the presentation of the instrument and the taking of anthropometric measurements, while in the second intervention an evaluation of the muscle strength protocol was carried out at 20% of body weight.

The isometric muscle strength assessment session began with a general warm-up consisting of (a) 5 min on a stationary bike at 60% of heart rate reserve, followed by 5 min of joint mobility exercises for the shoulder, elbow, wrist, and knee, and (b) a specific warm-up consisting of 3 sets of 5 s (s) each with 30 s of rest between sets, using a load of 15% of their body weight for each muscle strength assessment. The familiarization sessions were submaximal and were excluded from the analysis.

**Figure 1:**
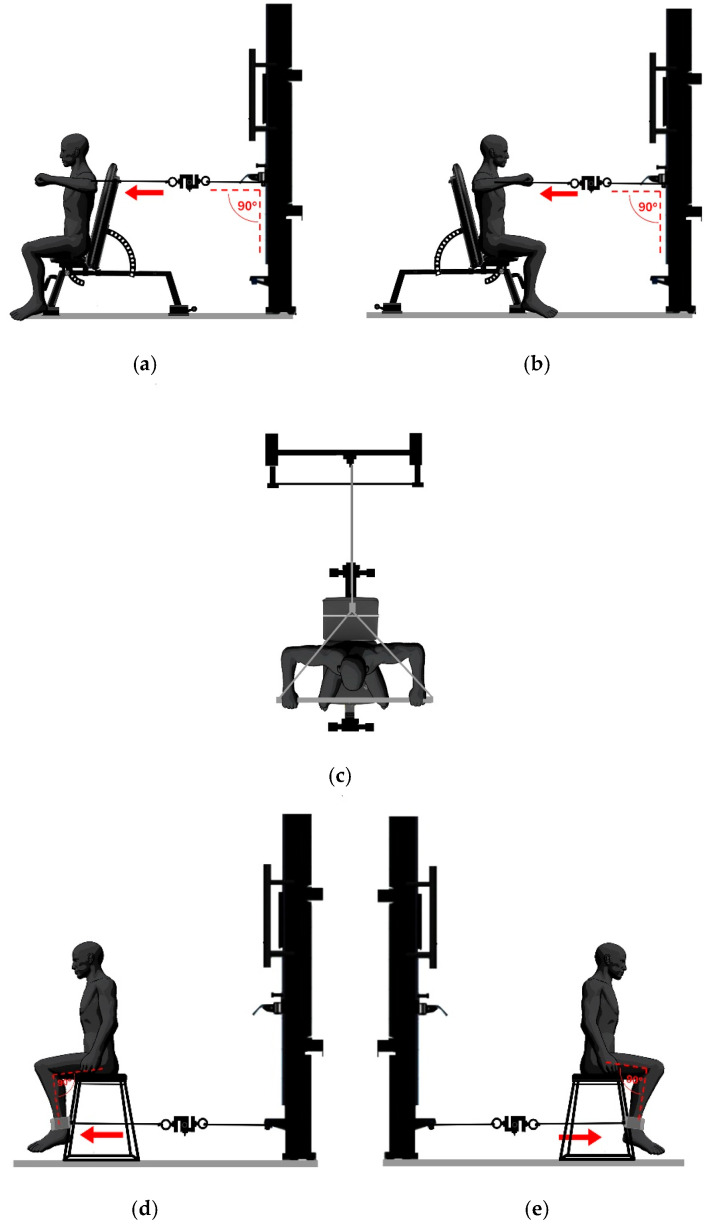
Position for muscle strength assessment: (**a**) bilateral seated bench press with shoulder abduction to 90° and elbow flexion to 90°, (**b**) bilateral seated row with shoulder abduction to 90° and elbow flexion to 90°, (**c**) horizontal plane view bilateral grip, (**d**) seated unilateral left knee extension in open kinetic chain with knee flexion to 90°, (**e**) seated unilateral right knee extension in open kinetic chain with knee flexion to 90°.

The assessment of maximal isometric muscle strength was performed in a seated position. Participants were asked to exert maximum effort during the measurements. BSBP and BSR assessments were performed bilaterally with shoulders and elbows at 90°, while USKER and USKEL assessments were performed unilaterally with the knee joint at 90°. Two evaluation sessions were conducted with an interval of 48 to 72 h. For each measurement, 3 sets of 5 s were performed with 3 min of rest between them until the maximum isometric strength was reached in each of the evaluations, selecting the best trial for subsequent analysis, as shown in Figure 1.

### 2.5. Statistical Analysis

Measures of central tendency (mean) and standard deviation (SD) dispersion measures were used to report descriptive statistics for the variables. Data distribution was analyzed using the Shapiro–Wilk statistical model (*p* > 0.05). The paired *t*-test and effect size (ES) of standardized mean differences for repeated samples were used to compare the magnitude of isometric muscle strength between two assessment sessions. The criteria used to interpret the magnitude of the ES were those described below: very large (>2.00), large (1.20–2.00), moderate (0.60–1.19), small (0.2–0.59), and null (<0.20) [25]. Absolute reliability was measured using the standard error of measurement (SEM), and the coefficient of variation (CV) was also included in the analysis. Relative reliability was measured using the intraclass correlation coefficient (ICC) 1.1 statistical model [26]. The following criteria were used to categorize absolute reliability: high reliability (CV ≤ 5%) and acceptable reliability (CV ≤ 10%) [27]. Relative reliability (ICC) was classified as values close to 0.9(extremely high reliability), 0.7 (very high), 0.5 (high moderate), 0.3 (low) and 0.1 [28]. Bland–Altman graphical statistical models were used to quantify systematic bias and 95% limits of agreement between test and retest [29]. The coefficient of determination was used to analyze the heteroscedasticity of the errors in the Bland–Altman charts (R^2^ > 0.1) [30]. Statistical significance was accepted with a *p*-value < 0.05, the 95% confidence intervals reported in Table 1 correspond to the ICC estimates and were calculated using the ICC(1,1) model in observations JASP software (version 0.16.4), with the participant as the unit of analysis (n = 18); repeated measurements within each participant were not treated as independent.

## 3. Results

For the evaluation of muscular strength isometric, no significant differences were observed (*p* > 0.05) and an ES classified as null (ES < 0.12) was detected in the BSBP, BSR, USKER and USKEL measurements. In the inter-session reliability measurements, acceptable absolute reliability was presented for BSR and USKER, extremely high reliability for the BSBP and USKEL measures, and extremely high relative reliability was reported for all assessments of maximum isometric muscle strength, as presented in Table 1.

**Figure 2:**
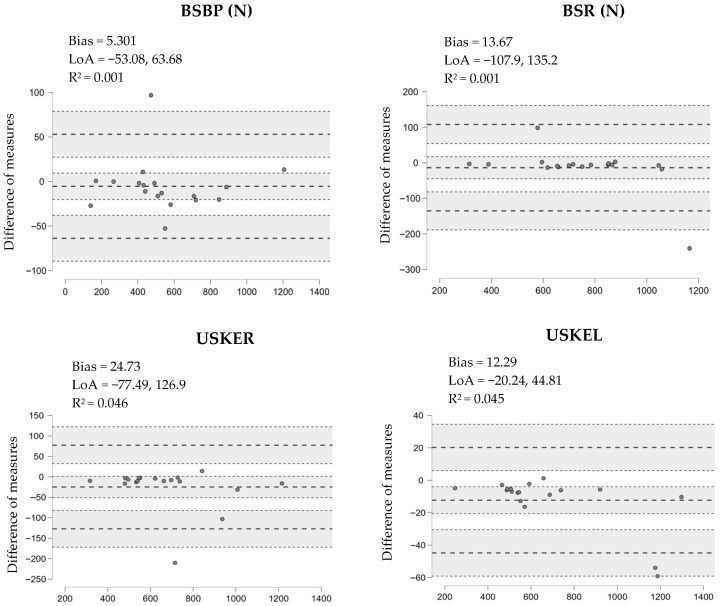
Test–retest Bland–Altman plots for isometric maximal muscle strength bilateral seated bench press (BSBP), bilateral seated row (BSR), unilateral seated right knee extension (USKER), and unilateral seated left knee extension (USKEL) using the Chronojump Force Sensor Kit.

Bland–Altman plots with a positive bias (5.301–24.73 N) are reported for the intersession assessment of maximal isometric muscle strength in bilateral seated bench press (BSBP), bilateral seated row (BSR), seated unilateral right knee extension (USKER), and seated unilateral left knee extension (USKEL). The coefficient of determination of the different measurements is presented between R^2^ = 0.001–0.046, as shown in Figure 2.

## 4. Discussion

The purpose of this study was to analyze the absolute and relative reliability between sessions of an isometric assessment of muscle strength in an older adult population. This study reported acceptable reliability for the BSR and USKER assessments, and extremely high reliability for the USKEL and BSBP assessments. Furthermore, extremely high relative reliability was reported for all assessments of maximal isometric muscle strength, with no significant differences observed between the two sessions of maximal isometric strength measurement.

Our study obtained consistency with the study Shahidi et al. [31], which analyzed the reliability of a new device (Powrlink), in a population of active university students, observing a high relative reliability (ICC = 0.99) in the assessment of maximum isometric muscle strength, which supports its reliability for accurately assessing knee extensor strength. In the study published by Sangkarit et al. [32], the Nintendo Wii Balance Board was used to assess isometric strength in the knee extensor muscles of older adults. The reliability measures presented between the two muscle strength assessment sessions yielded an ICC = 0.98. While these results are considered adequate for this population, the measurement device was not designed for this purpose, which is reflected in a larger absolute error and some limited sensitivity for detecting small changes between sessions. In contrast, in our study using the Chronojump Force Sensor Kit, similar reliability values were obtained, and the assessment of leg extensors was higher (ICC = 0.99), indicating much more robust stability for our protocol. This difference is likely due to both the accuracy of the calibrated force sensor and the strict control of the assessment conditions, especially posture and effort duration. Overall, although both studies confirm that isometric assessment is a useful and reliable tool in older adults, the results obtained with Chronojump show a greater ability to detect real changes in muscle strength, making it a more suitable method for clinical and sports applications, where a more accurate and consistent record is required.

In a study conducted by researchers Andrades-Ramírez et al. [33], the consistency of measurements for different manifestations of muscle strength was analyzed in physically active young adults. Participants performed seated bench press, seated row, and unilateral knee extension tests using a functional electromechanical dynamometer (FEMD), a device known for its accuracy and ability to operate in both isometric and isometric/vibratory modes. The authors reported acceptable absolute reliability (CV < 10%) and extremely high relative reliability (ICC = 0.92–0.99) for all muscle strength assessments, with no significant differences reported between the two assessment sessions and a negligible effect size. Similarities exist in the body position used for the strength assessment protocols, as both are performed seated, and the reliability of the test may be influenced by the stability of the seated position. In the study [34], the reliability of the IB-LS device was examined, which analyzed isometric muscle strength testing of the IB-LS device (InBody Inc., Seoul, Republic of Korea), obtaining a relative reliability ICC = 0.962–0.986, which provides stable and consistent muscle strength values in repeated intra-subject measurements. These results show that isometric muscle strength assessments have lower measurement errors and better reproducibility than some more conventional measurements of dynamic muscle strength, which reinforces the importance of ensuring consistency in repeated measurements in the assessment of muscle strength in any population and clinical or sports setting. Similar reliability results were obtained in study Gam et al. [35], in which most of the physical tests examined reported good to excellent reliability. Learning effects were also observed between sessions 1 and 2 as in our study, findings that clarify that learning effects can vary significantly between physical tests, even within the same cohort.

Future research should consider the limitations of this study. It is suggested that studies be initiated analyzing different populations of older adults, including those with metabolic diseases or hypertension, and those with eugenic diseases, as well as analyzing the reliability of other motor skills. It is noteworthy that mean values were consistently higher in repetitions of the different measures (Table 1), which could suggest a residual familiarization or learning effect despite the non-significant differences. The sample size could be considered small, but similar sample sizes have been used in studies with young, active men and female athletes [33,36].

## 5. Conclusions

The main results of this study demonstrate that maximal isometric muscle strength in bilateral seated bench press, bilateral seated row, and unilateral right and left knee extension, assessed using the Chronojump force sensor kit, is reliable and reproducible in older adults. A very easy-to-implement protocol is presented, utilizing an inexpensive and user-friendly device suitable for clinical and personal performance settings.

## Figures and Tables

**Table 1:** Relative and absolute reliability for 5 s maximal isometric muscle strength.

	Mean ± SD (N)						*p*-Value	ES	SEM	CV%	ICC
	Test			Re-Test					(95% CI)	(95% CI)	(95% CI)
BSBP	540.94	±	282.01	546.25	±	282.81	0.46	0.02	21.51 (16.02–32.74)	3.96 (2.95–6.02)	0.99 (0.98–0.99)
BSR	741.75	±	262.13	755.41	±	288.08	0.36	0.05	45.20 (33.66–68.79)	5.04 (4.50–9.19)	0.96 (0.90–0.98)
USKER	660.17	±	258.08	684.90	±	269.99	0.06	0.09	37.82 (28.17–57.56)	5.62 (4.19–8.56)	0.97 (0.92–0.98)
USKEL	670.08	±	312.53	682.37	±	323.06	0.05	0.04	37.82 (08.90–18.19)	1.77 (1.32–2.19)	0.99 (0.98–0.99)

BSBP: bilateral seated bench press; BSR: bilateral seated row; USKER: Unilateral seated knee extension right; USKEL: Unilateral seated knee extension left; N: Newtonss; *p*-value: significance level; SD: standard deviation; ES: Cohen’s d effect size; SEM: standard error of measurement; CV%: coefficient of variation; ICC: intraclass correlation coefficient; 95% CI: 95% confidence interval.

## Data Availability

The raw data supporting the conclusions of this article will be made available by the authors on request.
